# Covalent Probes To Capture *Legionella pneumophila* Dup Effector Enzymes

**DOI:** 10.1021/jacs.4c08168

**Published:** 2024-09-17

**Authors:** Max S. Kloet, Rishov Mukhopadhyay, Rukmini Mukherjee, Mohit Misra, Minwoo Jeong, Cami M. P. Talavera Ormeño, Angeliki Moutsiopoulou, Rayman T. N. Tjokrodirijo, Peter A. van Veelen, Donghyuk Shin, Ivan Đikić, Aysegul Sapmaz, Robbert Q. Kim, Gerbrand J. van der Heden van Noort

**Affiliations:** † Department of Cell and Chemical Biology, Leiden University Medical Centre, 2333 ZC, Leiden, The Netherlands; ‡ Buchmann Institute for Molecular Life Sciences, Goethe University Frankfurt am Main, 60438, Frankfurt am Main, Germany; § Department of Systems Biology, College of Life Science and Biotechnology, Yonsei University, 03722, Seoul, Republic of Korea; ∥ Centre for Proteomics and Metabolomics, Leiden University Medical Centre, 2300 RC, Leiden, The Netherlands

## Abstract

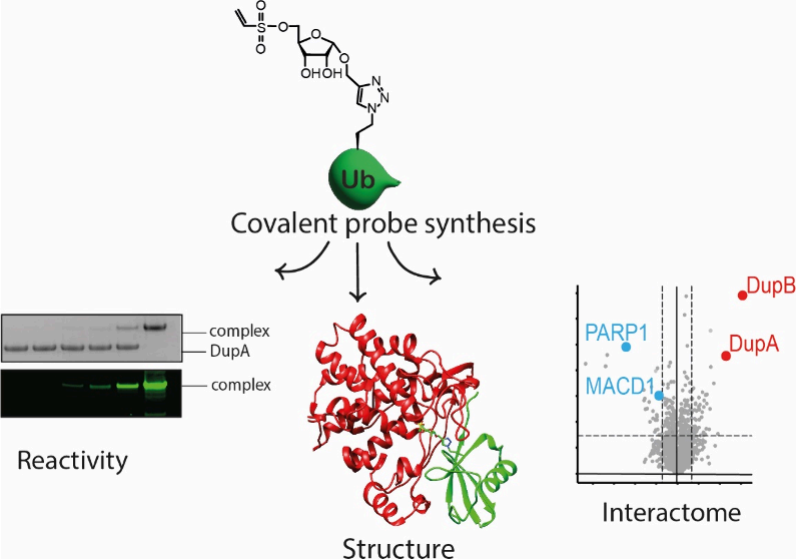

Upon infection of host cells, *Legionella pneumophila* releases a multitude of effector enzymes into the cell's cytoplasm that hijack a plethora of cellular activities, including the host ubiquitination pathways. Effectors belonging to the SidE-family are involved in noncanonical serine phosphoribosyl ubiquitination of host substrate proteins contributing to the formation of a Legionella-containing vacuole that is crucial in the onset of Legionnaires’ disease. This dynamic process is reversed by effectors called Dups that hydrolyze the phosphodiester in the phosphoribosyl ubiquitinated protein. We installed reactive warheads on chemically prepared ribosylated ubiquitin to generate a set of probes targeting these Legionella enzymes. In vitro tests on recombinant DupA revealed that a vinyl sulfonate warhead was most efficient in covalent complex formation. Mutagenesis and X-ray crystallography approaches were used to identify the site of covalent cross-linking to be an allosteric cysteine residue. The subsequent application of this probe highlights the potential to selectively enrich the Dup enzymes from Legionella-infected cell lysates.

## Introduction

Ubiquitination, a crucial post-translational modification (PTM) in eukaryotes, involves attachment of a 76 amino acid-long protein to substrate proteins. This modification plays a vital role in many cellular processes, including proteasomal degradation of proteins and the maintenance of genome stability.^1−3^ Adenosine diphosphate ribosylation (ADPr) is another PTM that is mainly known for its role in regulating DNA repair processes,^4−6^ but that also is widely recognized as signal for ubiquitination.^7^ As such, in addition to the individual impact of both these isolated PTMs on protein signaling, there has been a growing interest in the regulation of ubiquitination by ADPribosylation and vice versa.^8^ This interest has been particularly fueled by the discovery of a class of multidomain Legionella pneumophila effector enzymes that physically link these two post-translational modifications together en-route to effectively replicate within the host cell.^9,10^ The eukaryotic ubiquitination process begins with the activation of the C-terminus of ubiquitin by an E1 enzyme, which requires energy in the form of ATP. Following the formation of a reactive thioester linkage between E1 and Ub, ubiquitin is subsequently transferred to the E2 and finally, E3 enzymes ligate Ub onto mostly the lysine of target proteins via formation of an iso-peptide bond. In contrast, the ubiquitination mechanism used by Legionella SidE effectors in noncanonical phosphoribosyl (PR)-ubiquitination starts with ADP-ribosylation of Arg42 of ubiquitin in an NAD^+^ dependent manner, mediated by the mART-domain of SidE enzymes.^9,10^ The formed Ub^ADPr^ is subsequently transferred to the phosphodiesterase (PDE) domain of SidEs, which mediate the conjugation to a serine amino acid residue in target proteins resulting in a phosphoribosyl (PR) linkage between ubiquitin and the modified protein substrate (Supplementary Figure 1).^10−13^ Proteome analysis of Legionella infected cells identified proteins predominantly involved in Endoplasmatic Reticulum (ER) fragmentation and membrane recruitment to the Legionella-containing vacuole (LCV) to be PR-ubiquitination targets.^14,15^ Legionella hence gains local control over part of the host-ubiquitinome and is able to orchestrate efficient LCV formation in order to create a favorable environment for bacterial replication. Legionella depends on the SidE effector activities to proliferate inside the host cell, as bacterial replication is significantly hindered without these effectors.^9,16,17^ This process is highly dynamic and SidE PR-Ub ligase activity is regulated by glutamylase SidJ, and Ub^ADPr^ formation can be reversed by effector MavL.^18^ In addition deconjugation of PR-ubiquitination from the host cells proteins by deubiquitinases for phosphoribosyl ubiquitination (Dups) has been identified to control PR-ubiquitination levels.^14,17,19−21^ These Dups (DupA and DupB, also named LaiE and LaiF) consist of a single PDE-like domain and perform their action by directly cleaving the phosphomonoester linkage, restoring the native host protein and concomitantly releasing phosphoribosyl ubiquitin (Ub^PR^). This molecule has been speculated to locally inactivate the conventional host ubiquitination process and acts as an autophagic blockade, a process that is shown to in turn be reverted by LnaB activity that restores Ub^ADPr^.^14,19,22,23^ Although recent advances in the development of chemical tools, such as small molecule inhibitors,^24^ Ub^ADPr^ substrates^25^, stabilized Ub^ADPr^-analogues^26^ and fluorescence-polarization assay-reagents^27^ have demonstrated their value in the study of the PR-ubiquitination pathway, covalently binding probes frequently used to target conventional (de)ubiquitinating enzymes, have not been developed so far to target the PR-ubiquitination machinery. The majority of the tools currently available are used on recombinant proteins in isolation, and being able to study Legionella effectors in a more complex environment such as infected cell lysate could be a valuable addition.

In this work, we use an adaptable platform based on the copper catalyzed click reaction between an azide modified synthetic Ub protein and a variety of propargyl modified ribose molecules that are equipped with reactive groups to generate a small library of probes to covalently capture the Legionella Dup family. This method not only gives easy access to a variety of covalent probes but also results in a hydrolytically stable triazole bond between the Ub protein and ribose group, facilitating the application of the probes in cellular environments. The hence-created set of probes is subjected to recombinantly expressed DupA to verify covalent binding of the probes, and the site of reactivity is investigated using mutagenesis and structural approaches. Subsequent application of the most reactive probe in pull-down experiments from Legionella infected HEK293T cell lysate shows excellent enrichment of the targeted Legionella enzymes, highlighting the applicability of our probes to study Legionella effector enzymes in complex biological settings.

## Results and Discussion

### Chemical synthesis allows introduction of diverse warheads onto Ub

**Figure 1:**
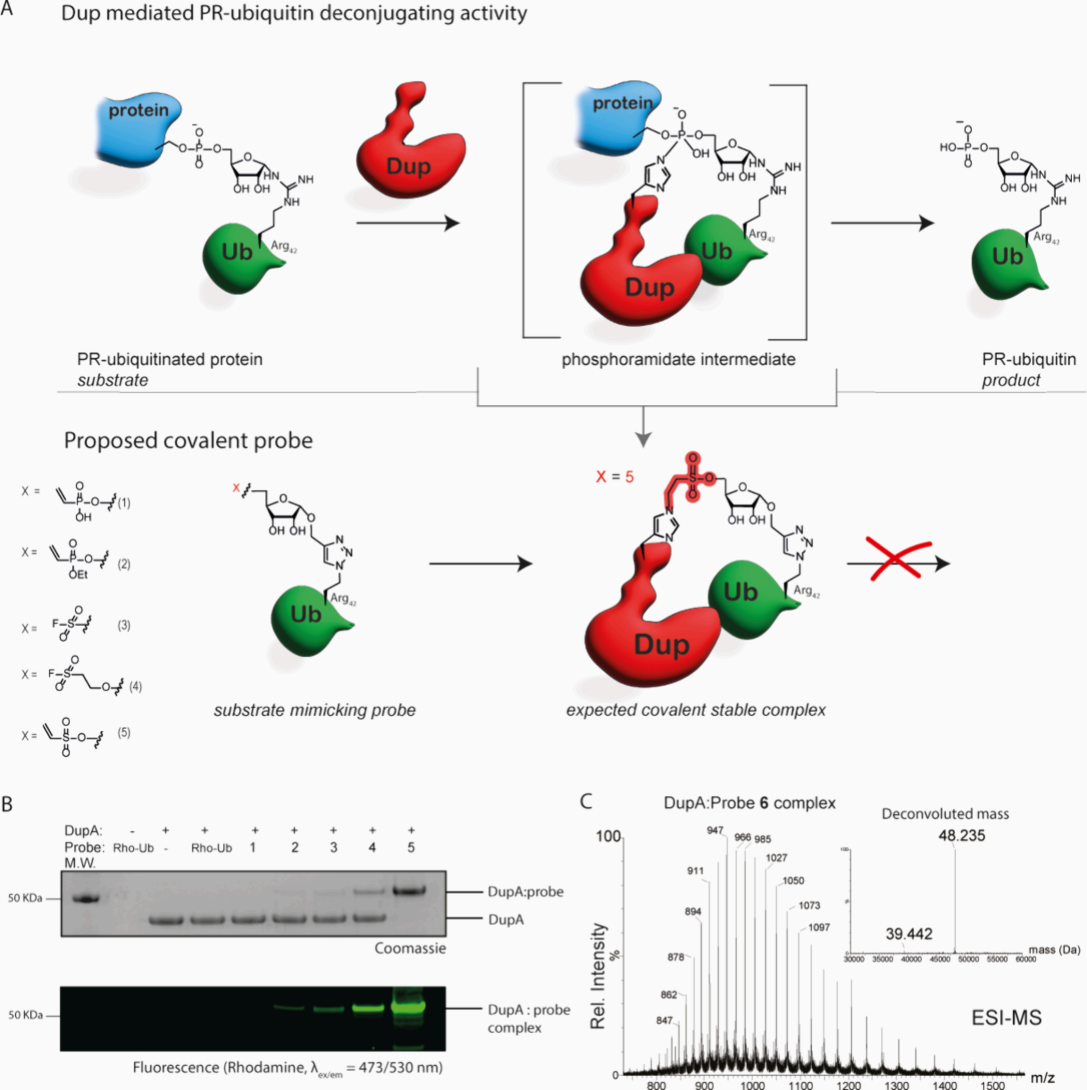
Assessment of the reaction between probe library **1**–**5** and recombinant DupA WT. (A) Schematic representation of part of the catalytic mechanism of Dup activity showing the covalent phosphoramidate intermediate that Dup-His67 forms when deconjugating a Ser-PR-ubiquitinated substrate (upper panel) and the expected chemical trapping of the covalent transition state intermediate by our substrate mimicking probe (lower panel). The different warheads installed on probes **1**–**5** are depicted on the left. The stable covalent complex formed impedes hydrolysis to the PR-Ub product. (B) SDS-PAGE gel showing complex formation between probes **1**–**5** with DupA WT. Probe **1**–**5** (8 equiv) were subjected to DupA and incubated at 37 °C for 2 h and analyzed by SDS-page: Coomassie stain (upper panel), Rhodamine fluorescence scan (λ_ex/em_ = 473/530 nm, bottom panel). (C) Mass spectrometry analysis of DupA:probe **6** complex, ESI MS of the conjugate reacting vinyl sulfonate probe **6** (deconvoluted mass: 8.793 Da) to DupA WT (deconvoluted mass: 39.442 Da). Deconvoluted mass of complex (found: 48.235 Da, calculated: 48.237 Da) confirming the covalency of the conjugate.

Both the catalytic domains of SidE ligases (PDE-domain) and the deconjugating Dups rely on a triad of Glu-His-His in contrast to the cysteine-based catalysis present in most conventional Ub-ligases and -proteases.^28^ It has been proposed that during cleavage of the phosphodiester bond, the Dups form a covalent phosphoramidate intermediate initiated by nucleophilic attack of His67 on the PR-ubiquitinated substrate thereby liberating the native protein (Figure 1A – upper panel). The subsequent step in the hydrolysis reaction is facilitated by the second reactive histidine of the Dup (His189) that activates a water molecule to attack and hydrolyze the formed Dup(His67)-PR-Ub intermediate (Supplementary Figure 2). The formation of the initial covalent intermediate between the Dup and its substrate opens the opportunity to design covalent probes in analogy to probes carrying Michael acceptor type warheads such as Ub-VME or Ub-PA, used to capture cysteine deubiquitinating enzymes (DUBs).^29,30^ To covalently trap the two members of the Legionella Dup family, we prepared a set of probes that carry a reactive group substituting the phosphodiester group that in a bonafide substrate links the serine substrate protein to PR-Ub. Upon nucleophilic attack of the Dups’ active site His67 to the probe, a stabile intermediate that cannot be further hydrolyzed by the enzyme will result in a permanent covalent bond between probe and enzyme (Figure 1A – lower panel). However, targeting catalytic histidine residues is challenging, as the nucleophilicity of this residue is moderate compared to lysine or cysteine. Among others, warheads such as fluorosulfonates,^31,32^ vinyl sulfonates,^33^ vinyl phosphonates^34^ and thiophosphorochloridates^35^ were shown capable to covalently bind surface exposed histidines, although examples of active site histidine reactivity are scarce.^36,37^ Five warheads tailored to react with the mild nucleophilic histidine residue were selected and installed at the 5′-hydroxyl of the ribosyl group: vinyl phosphonate **1**, vinyl ethoxy phosphonate **2**, fluorosulfonates **3** and **4** and vinyl sulfonate **5**. After synthesis of the ribosides (Supplementary Schemes 1 and 2) equipped with the diverse warheads at the 5′-hydroxyl and a propargyl moiety installed at the 1′-hydroxyl we used copper catalyzed click chemistry to conjugate full length ubiquitin carrying a rhodamine fluorophore on the N-terminus and an Arg42 to azido-homoalanine mutation to each of the ribosides, resulting in five fluorescently labeled covalent probes (Figure 1A – lower panel).^38^

### Chemical probes react covalently with recombinantly expressed DupA

The reactivity of probes **1**–**5** was evaluated by incubating them with recombinantly expressed DupA for 2 h at 37 °C (Figure 1B). Both vinyl phosphonate **1** and its ethoxy variant **2** showed no or only little complex formation based on in-gel fluorescence and Coomassie staining, in-line with literature reports indicating such vinyl phosphonates to be low in reactivity.^34^ Additionally, fluorosulfonates **3** and **4** showed moderate complex formation, with the longer spacer probe **4** showing slightly better results than its shorter analogue **3**. Gratifyingly, the vinyl sulfonate probe **5** quickly reacted and reached full conversion even after shorter incubation times of only 15 min (Supplementary Figure 3). To further investigate the labeling efficiency of probe **5**, we performed a time series on ice to get an indication of the rate of binding of our probe, observing close to full conversion as soon as 5 min when using 5 equiv of probe (Supplementary Figure 4A). We hence concluded the vinyl sulfonate warhead used in probe **5** resulted in our most reactive probe, and we applied this warhead in the rest of our studies. To verify the covalent nature of the formed complex, we performed high resolution intact protein MS analysis using a nontagged version of the vinyl sulfonate probe (**6**), to exclude influence of the rhodamine dye on the binding event. The deconvoluted mass of the formed complex (*m*/*z* = 48.235 Da) corresponds to the calculated mass of the DupA:probe complex and verifies the covalent nature of the linkage (Figure 1C). To provide a first indication of selectivity of our probe to Legionella effector enzymes, we tested the reactivity of the vinyl sulfonate **5** to conventional cysteine family deubiquitinating enzymes OTUB2, UCHL3 and USP21 and found no reactivity toward these canonical DUBs. Additionally, NAD^+^ consuming enzymes NMNAT1 and ART1 were examined and probe **5** was unable to covalently react with either of these enzymes (Supplementary Figure 5). We then explored the significance of the triazole linkage in probe binding by synthesizing a guanidinium-linked variant **7** using previously established methods.^25^ We prepared an additional Ub-probe **8** where we installed the vinylsulfonate warhead onto Ub via an alkyl linker directly. A comparison of the binding efficiencies between compounds **5**, **7** and **8** on recombinant protein (Supplementary Figure 4) indicates a swift complex formation with DupA and a comparable reactivity, indicating that the linker region does not significantly impact binding to DupA (Supplementary Figure 4C).

### DupA mutational analysis reveals unexpected site of reactivity

**Figure 2:**
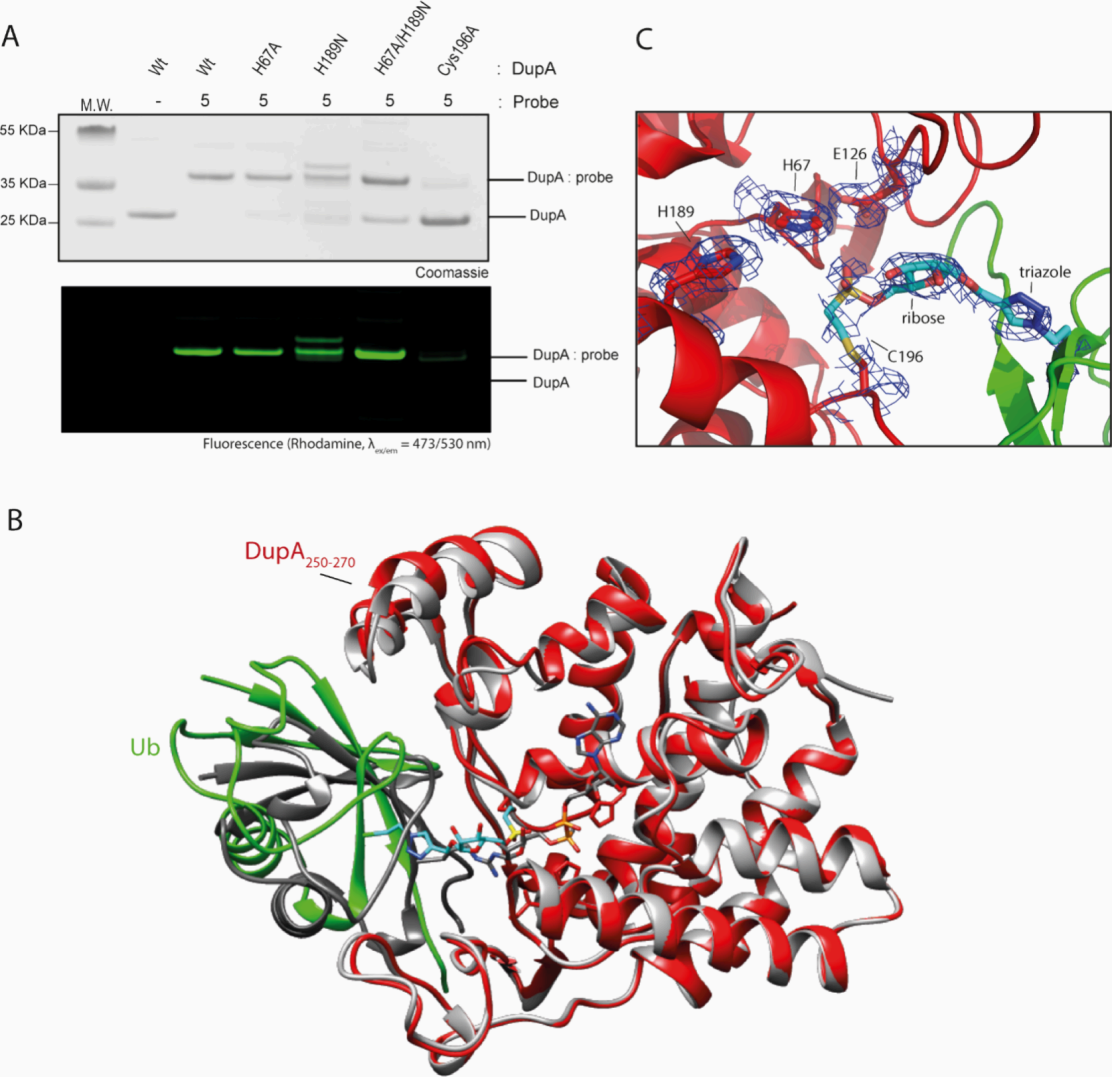
Mutagenesis and crystallographic investigation of the covalent DupA:probe complex. (A) Mutagenesis of DupA and reactivity of the mutants to rhodamine labeled vinyl sulfonate probe **5**. (B) Overview of the DupA:probe **6** complex crystal structure (in red and green respectively; PDB: 9EMK), compared to DupA-His67Ala:Ub^ADPr^ complex (PDB: 6B7O; in gray and dark gray respectively). (C) Structure and electron density of the cross-linked region (cyan) between DupA (red) and Ub probe **6** (green).

To confirm that our PR-Ub probe **5** acts as a suicide inhibitor, we first incubated DupA with probe **5** to form a complex, followed by the addition of Ub^ADPr^, which is a DupA substrate. DupA activity was visualized using mass spectrometry analysis by the appearance of Ub^PR^ due to hydrolysis of the pyrophosphate bond in substrate Ub^ADPr^. Indeed, Ub^ADPr^ was not cleaved by the DupA:probe **5** complex, whereas in the absence of preincubation with probe **5**, Ub^ADPr^ hydrolysis mediated by DupA is swiftly observed (Supplementary Figure 6). This indicates that probe **5** abrogates the catalytic activity or blocks the active site. In order to pinpoint the exact site of covalent modification, we performed mutational analysis, where we mutated His67 or His189 and unexpectedly observed a retained reactivity of both mutants to probe **5** (Figure 2A). We speculated that when one of the active site histidines was mutated the probe might react with the remaining histidine, that is situated in close proximity. However, even the DupA double mutant (His67Ala/His189Asn) was significantly labeled by probe **5**. When examining the catalytic triad of DupA, the crystal structure of Ub interacting with DupA (PDB: 6RYA) revealed that Cys196 resides in the same helix as His189 and is in close proximity to both active site His67 and His189. Although Cys196 is not part of the catalytic triad and is placed slightly outside of the active site, we wondered whether this cysteine might react with probe **5**, as the vinyl sulfonate warhead is a Michael acceptor that could also react with cysteine. Indeed, despite some minor residual labeling activity, reacting probe **5** with the Cys196Ala mutant significantly reduced the formation of the DupA–probe complex. The Cys196Ala mutant however was fully active in cleaving Ub^ADPr^ to Ub^PR^ (Supplementary Figure 7). Treating DupA with a cysteine-alkylating agent (iodoacetamide) prior to incubation with probe **5** abrogates labeling (Supplementary Figure 3C); likewise ADPr to PR processing is inhibited by iodoacetamide treatment (Supplementary Figure 7). These results indicate that Cys196 is not crucial for catalysis per se, but introducing steric bulk blocks substrates from entering the active site cavity.

Next we subjected the formed DupA:probe **5** complex to trypsin digestion, followed by tandem MS analysis. When comparing nontreated DupA WT versus DupA:probe **5** complex a change in peptide fragments identified was observed (Supplementary Figure 8). The tryptic peptide fragments that were absent in the latter case might indicate probe **5** covalently cross-linking itself to an amino acid residue in that particular peptide thereby modifying it and hindering its identification. Indeed, the tryptic peptide fragment harboring Cys196 (C196 K197) was observed for DupA WT and was no longer present after reacting with probe **5**. No alterations were observed in the peptide coverage of both the regions harboring the active site His67 or His189 residues. The peptide belonging to the Ub-remnant (Ub_34–48_) cross-linked to the DupA_196–197_ peptide was subsequently identified (Supplementary Figure 9).

### X-ray crystallography confirms reactivity to Cys196

To further consolidate the findings that probe **5** covalently reacts with Cys196 of DupA, we formed the complex between DupA and probe **6** (the nontagged version of probe **5**) and were able to obtain diffracting crystals (PDB: 9EMK) (Supplementary Table 1). With the *P*3_2_ space group and the inability of direct molecular replacement with PDB model 6RYA(14) (space group: *C*2), we had to resort to molecular replacement using apo DupA (PDB: 6RYB) and manually place Ub (PDB: 1UBQ) to determine the structure. This still yielded an asymmetric unit with 3 DupA complexes, albeit differently arranged through contacts. Upon closer inspection, the DupA monomer compares very well to those in structures of DupA-apo (PDB: 6RYB), DupA^His67Ala^-Ub (PDB: 6RYA) and DupA^His67Ala^-Ub^ADPr^ (PDB: 6B7O) with RMSD values <0.4 Å (Supplementary Figure 10A). The main difference is in the DupA α-helical lid (a.a. 250–270) that protrudes slightly and makes contact with the bound ubiquitin (Figure 2B). Indeed, when comparing the ubiquitin moiety between the Ub-bound structures (6RYA, 6B7O) we can observe a small tilt and movement for Ub probe **6**, making a more snug interaction with the aforementioned loop_250–270_. The most likely reason for this movement is found at the binding site, where the reactive warhead has inserted itself toward active site His67 (Supplementary Figure 10B), but is caught and bound by Cys196 before fully reaching the target (Figure 2C). This creates some steric repulsion because of the local strain in the sulfonate probe when ubiquitin would try and nest itself in the actual binding site, so instead, it now binds loop_250–270_, albeit with some flexibility. This flexibility is also seen in the associated B-factors as well as how well the connecting warhead is defined in the electron density. The found density nevertheless further confirms our MS-MS data (Supplementary Figures 8 and 9) and mutational analyses (Figure 2A) and shows that the vinylsulfonate probe binds Cys196 of DupA.

### The vinylsulfonate probe is able to enrich both DupA and DupB from Legionella infected HEK293T cell lysate

**Figure 3:**
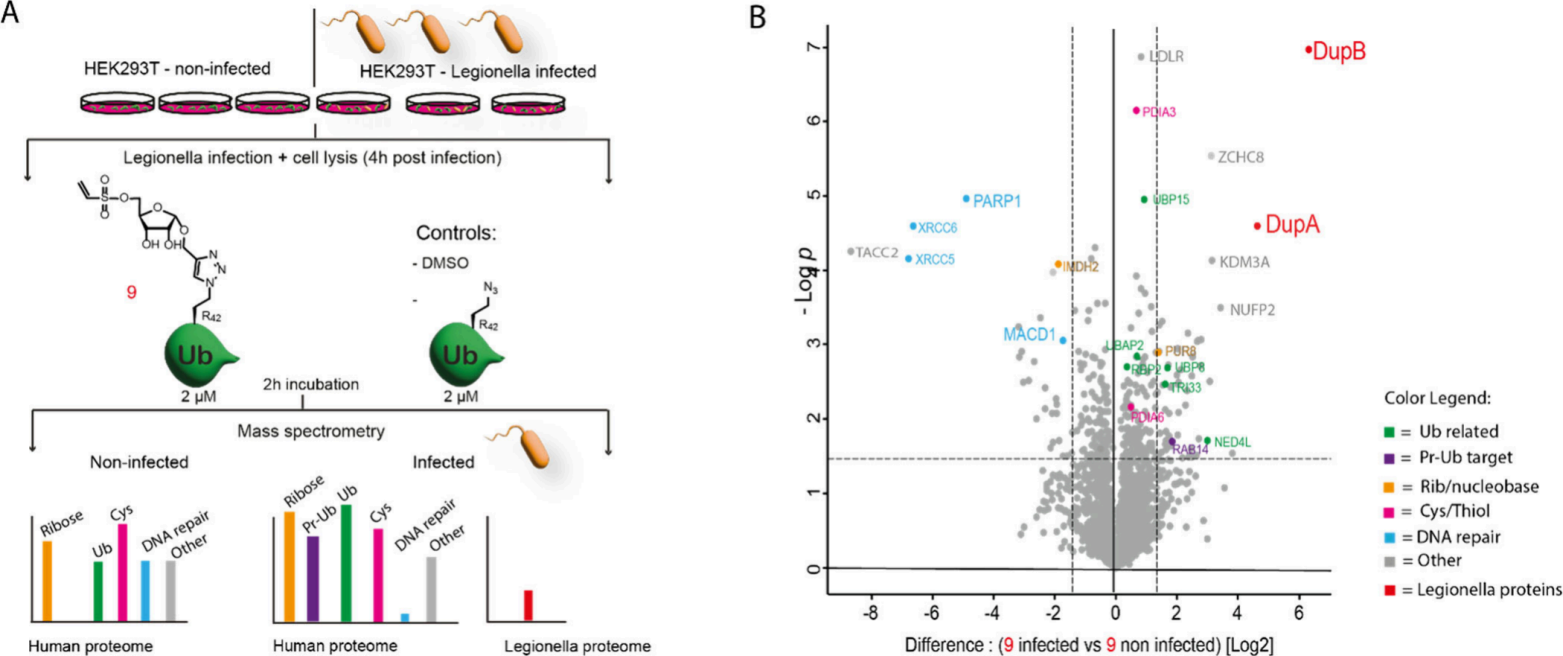
Chemoproteomic assessment of pulldown by the vinylsulfonate probe **9** from noninfected HEK293T cells or cells infected with Legionella. (A) Schematic representation of the workflow applied in mass spectrometry-based proteomics. The lysates of two sample groups; infected with Legionella and noninfected, were prepared in triplicate. (B) Volcano plot depicting proteins enriched by probe **9** comparing the infected sample group to the noninfected sample group (dotted line represents significance and correspond to the thresholds: log_2_ ratio ≥1.5; *p*-value ≤0.05. A color code legend is provided for the clustered proteins. The Legionella enzymes DupA and DupB are marked in red.

As probes **5** and **6**, both equipped with the vinylsulfonate warhead, label recombinant DupA with high reactivity, we set out to validate the potential of such a probe to enrich Dups from the lysate of human HEK293T cells infected with Legionella. To facilitate the pulldown and allow for efficient visualization in the optimization process, we prepared vinyl sulfonate Ub probe **9**, a probe similar to **5** (and **6**) but equipped with both a rhodamine fluorophore and biotin-moiety. We used azido-Ub (the Arg42 to azido-homoalanine mutant, not functionalized with the vinysulfonate ribosyl moiety) and DMSO treatment as controls (Figure 3A). Both noninfected HEK293T cells and cells infected with Legionella were lysed 4 h postinfection as the optimal time point for detecting the activity of the Dups.^14^ The lysates were incubated with probe **9** or the appropriate controls at 37 °C for 2 h (optimization shown in Supplementary Figure 11). Neutravidin beads were added to capture the biotin-Ub-probe-protein conjugates. After stringent washing with buffer containing 2% SDS to remove all noncovalently interacting proteins, the beads were subjected to elution followed by trypsin digestion and mass-spectrometric analysis of the proteome. When comparing proteins enriched by probe **9** in the infected cells compared to noninfected cells, both DupA and DupB are significantly enriched (Figure 3B). When comparing probe **9** and the biotin-Ub control within the infected sample group, DupA and DupB again show significant enrichment (Supplementary Figure 12). Comparing biotin-Ub versus DMSO (Supplementary Figure 12) showed no significant enrichment of either Dup, highlighting the necessity of the warhead leading to the formation of the covalent complex between the probe and the Dups under Legionella infected conditions as well as underscoring that noncovalent interaction with Ub is not enough for enrichment of these Dups. The use of the vinylsulfonate probe warrants covalent cross-linking to a significant number of proteins in the lysates, and although some of these captured proteins may be off-targets due to the high reactivity of the warhead, the intended Dup family is among the most significant enriched proteins. Overall, probe **9** shows remarkable specificity toward DupA and DupB since they were the only two specifically enriched among the 2930 distinct proteins in the Legionella proteome.^39^ The intensity of proteins cross-linked to the probe in lysate seems to increase in time, and hence a shortened incubation time might also warrant a decreased amount of identified off-targets (Supplementary Figure 13). We continued and classified the identified mammalian proteins in different clusters, based on their respective roles in regulating Ub dynamics (green), previously reported SidE-targets annotated to be PR-ubiquitinated (purple), potential reactivity toward the warhead (pink) and recognition of structural elements of the probe (orange) (Figure 3B, Supplementary Figure 12). Since our probe has a reactive warhead that captures nucleophilic amino acid residues, we are not surprised to find proteins that potentially recognize either the Ub-core or the phosphoribosyl-mimic attachment as substrate and subsequently covalently react with the probe during the 2 h incubation period. Ub-conjugating enzymes like NEDD4L and TRIM33 and Ub proteases, including ubiquitin-specific proteases (USPs)—USP3, USP14, USP48, OTUB1 and USP15—were among the enriched human proteins that show reactivity toward the probe (Supplementary Figure 12, Figure 3B). The activity of the conventional ubiquitin machinery is known to be manipulated by bacterial infection, and therefore, it might be interesting to further investigate these specific enriched Ub-ligases and DUBs that show a change in reactivity toward our probe upon infection by the Legionella bacterium (in green; Figure 3B). We further annotated proteins previously implicated to be PR-ubiquitination targets in purple.^14,19^ Some of these also include Ub conjugating enzymes UBR5 and UBA1 and deubiquitinases such as USP5 and USP10, but we also enrich MAP4, CCAR2, PRDX1, ACLY, ATXN2L and EIF3D and several Rab GTPases—RAB14 (Figure 3B), RAB5C, RABL6 and RAB21 (Supplementary Figure 12), some of which are known substrates for PR-ubiquitination. These GTPases are involved in membrane trafficking and/or the endocytic pathway, and by manipulation of these proteins the bacterium might try to improve stability of the LCV.^40^

In addition, comparison of noninfected and Legionella infected cells leads to some interesting changes of protein reactivity toward our probe that might give insights in how the bacterium manipulates the host cell (Figure 3B). We observed significant enrichment for mammalian PARP1 and MacroD1, but interestingly in the left panel, indicating a marked decrease of protein levels and/or reactivity toward the probe during Legionella infection compared to noninfected cells. Indeed, probe **9** labels recombinant PARP1 efficiently and does not rely on cysteine reactivity, as iodoacetamide treatment did only slightly reduce reactivity of PARP1 toward the probe (Supplementary Figure 14A). Western-blot analysis shows the PARP1 protein levels as well as poly-ADPribose (PAR) formation in the sample to be lower in the infected lysate compared to the control lysate (Supplementary Figure 14B). PARP1 has previously been described to play a role in the immune response during *Salmonella enterica* and *Serovar Typhimurium* infection where it regulates NF-κB-mediated pro-inflammatory gene expression.^41,42^ The role of PARP1 during Legionella infection, however, has not been clarified to date, and decreasing activity of PARP1 by the bacterium potentially could affect the initiation of the immune response during infection. Another notable observation is the enrichment of DNA damage response proteins XRCC5 and XRCC6 in the left panel (Figure 3B).^43,44^ XRCC5 and XRCC6 are shown to be essential in regulation of the innate immunity response upon viral infections, and it could therefore well be that in analogy, Legionella acts on these proteins to suppress the host immune response.^45^ Additionally, PARP1 and XRCC5 as well as XRCC6 have been identified prior as potential PR-ubiquitination targets.^19^

## Discussion

Legionella manipulates its host cell by releasing a wide variety of effector enzymes. Among these effectors, the SidE enzyme family plays a crucial role in deregulating Ub signaling by effecting PR-ubiquitination, thereby favoring the replication of Legionella in the Legionella-containing vacuole. This PR-ubiquitination process itself is tightly regulated by SidJ enzymes and reversed by Dup effectors. Measuring or interfering with these different enzyme activities will help to gain fundamental molecular insights into these crucial steps in Legionella infection. We hence developed a chemical methodology for the generation of a collection of ubiquitin phosphoribose analogues, which serve as covalent probes to target Legionella Dup effectors. We successfully generated a highly reactive vinyl sulfonate probe that can covalently react with recombinant DupA, achieving fast and complete conversion. Although this vinyl sulfonate warhead was initially designed to target the catalytic histidine of the Dups, our studies indicate the main reactivity of probe **5** toward the Cys196 residue. Structural analysis indeed confirmed covalent cross-linking of the noncatalytic Cys196 forming a stable link between DupA and the PR-Ub derived probe. Although the targeted cysteine is a noncatalytic residue, the location near the active site might make this residue an interesting target for future drug development as reacting this cysteine residue with not only the bulky Ub-based probe but also the small alkylating reagent iodoacetamide reduced catalytic activity. We further successfully enriched both DupA and DupB from Legionella-infected HEK293T lysate, in a pull-down proteomics experiment, with the protease stable triazole linked probe **9**, confirming the applicability of this probe in a complex proteome. Of note, we did not enrich the SidE effectors in this pulldown experiment, and although the PDE domains of SidEs have a high structural homology to the Dups, the Cys196 from the Dups is not conserved in the SidEs. In addition the Legionella-infected cells used in our study were lysed 4 h postinfection and previous research indicates that the activity of SdeA is concentrated in the initial period after infection and is significantly reduced 4 h postinfection.^46^ Recent interest in regulatory mechanisms involving ADPribosylation of Ub or vice versa both in bacteria^17,20,21,47−49^ and mammals^50−53^ has grown, and the covalent tools presented here could be adapted and applied in a broader context. Dup protein levels can, for instance, be monitored during the course of Legionella infection, and labeling of Dups by the probe forms a potential read-out in a screen for inhibitors of Dup activity. In addition, the covalent nature of the probe could facilitate discovery of orthologous proteins in, e.g., other intracellularly replicating pathogenic bacteria. Consequently, further investigation of the Ub and ADPr interplay in human cells and the utilization of the probes presented here could help to unveil the molecular mechanisms involved in both normal homeostasis as well as during bacterial infection conditions.^8^

## Supporting Information Available

Supporting Information includes supplementary figures, detailed assay protocols and chemical methods and analysis of probes and intermediates. (PDF)
